# Combined Ramipril and Black Seed Oil Dosage Forms Using Bioactive Self-Nanoemulsifying Drug Delivery Systems (BIO-SNEDDSs)

**DOI:** 10.3390/ph15091120

**Published:** 2022-09-08

**Authors:** Ahmad Abdul-Wahhab Shahba, Abdelrahman Y. Sherif, Ehab M. Elzayat, Mohsin Kazi

**Affiliations:** 1 Department of Pharmaceutics, College of Pharmacy, King Saud University, P.O. Box-2457, Riyadh 11451, Saudi Arabia; 2 Kayyali Chair for Pharmaceutical Industries, Department of Pharmaceutics, College of Pharmacy, King Saud University, Riyadh 11451, Saudi Arabia

**Keywords:** black seed oil, ramipril, BIO-SNEDDS, hypertension, combined delivery systems

## Abstract

**Purpose**: Ramipril (RMP)—an angiotensin-converting enzyme (ACE) inhibitor—and thymoquinone (THQ) suffer from poor oral bioavailability. Developing a combined liquid SNEDDS that comprises RMP and black seed oil (as a natural source of THQ) could lead to several formulations and therapeutic benefits. **Methods**: The present study involved comprehensive optimization of RMP/THQ liquid SNEDDS using self-emulsification assessment, equilibrium solubility studies, droplet size analysis, and experimentally designed phase diagrams. In addition, the optimized RMP/THQ SNEDDS was evaluated against pure RMP, pure THQ, and the combined pure RMP + RMP-free SNEDDS (capsule-in-capsule) dosage form via in vitro dissolution studies. **Results**: The phase diagram study revealed that black seed oil (BSO) showed enhanced self-emulsification efficiency with the cosolvent (Transcutol P) and hydrogenated castor oil. The phase diagram studies also revealed that the optimized formulation BSO/TCP/HCO-30 (32.25/27.75/40 % *w*/*w*) showed high apparent solubility of RMP (25.5 mg/g), good THQ content (2.7 mg/g), and nanometric (51 nm) droplet size. The in-vitro dissolution studies revealed that the optimized drug-loaded SNEDDS showed good release of RMP and THQ (up to 86% and 89%, respectively). Similarly, the isolation between RMP and SNEDDS (pure RMP + RMP-free SNEDDS) using capsule-in-capsule technology showed >84% RMP release and >82% THQ release. **Conclusions**: The combined pure RMP + RMP-free SNEDDS (containing black seed oil) could be a potential dosage form combining the solubilization benefits of SNEDDSs, enhancing the release of RMP/THQ along with enhancing RMP stability through its isolation from lipid-based excipients during storage.

## 1. Introduction

The demand for natural active/inactive formulation ingredients is growing due to their attractive characteristics, such as their ability to dissolve many active pharmaceutical ingredients as a result of their chemical composition and biodegradability. Plant-derived pharmaceutical ingredients are usually safer, as they produce fewer toxic metabolites. Various diseases (such as cancer, stroke, diabetes, atherosclerosis, and Alzheimer’s disease) have been safely managed with natural ingredients and antioxidant-based formulations [1]. Currently, bioactive natural oils play important roles in the development of new drug delivery systems—especially for psychoactive, antimicrobial, and anticancer agents. The exceptional pharmacological benefits of these ingredients attract scientists to explore and characterize their biological profiles. Such exciting ingredients increase the likelihood of obtaining new targeted therapies for several challenging diseases [2,3].

Uncontrolled hypertension can be treated with monotherapy or combination therapy. The former is usually undesirable by physicians because of the risk of side effects of high-dose monotherapy. Conversely, combination therapy avoids exposure to high doses of each therapeutic drug, with a desirable therapeutic response [4]. Amongst antihypertensive agents, angiotensin-converting enzyme inhibitors have superior benefits over other classes. This is attributed to their protective effects on the cardiovascular and renal systems [5]. Ramipril (RMP) is a potent angiotensin-converting enzyme inhibitor that has been widely used in controlling several diseases—e.g., hypertension, congestive heart failure—and to improve survival after a heart attack [6]. Side effects of RMP include postural hypotension, hyperkalemia, and angioedema [7]. These side effects are mainly dose-dependent; hence, combination therapy with a low dose of RMP is highly desirable to decrease side effects. In addition, the combination therapy adds synergistic effects to the dosage form.

In this context, black seed is considered to be one of the greatest forms of herbal medicine in several Islamic and Arabic countries. Black seed contains over 100 phytochemical constituents, which co-produce a synergetic effect supporting the immune system and strengthening the body’s constitution. A published review revealed that black seed and its extracted oil represent a multidisciplinary remedy that can successfully treat more than 129 diverse human ailments [8,9]. In particular, black seed oil (BSO) has a rich composition of several valuable components that play a vital role in forming prostaglandin (PG) E1, which balances and strengthens the immune system against infections, allergies, and chronic illnesses [8]. Many therapeutic properties of black seed have been suggested to be correlated with the presence of thymoquinone (THQ) within the essential oil [9]. In particular, black seed as a whole and its constituent THQ can play a protective role in a wide range of metabolic and cardiac diseases [10]. This is mainly due to its rich flavonoid and antioxidant contents. It has been observed in different studies that THQ efficiently improves cardiovascular diseases by decreasing β-hydroxy β-methylglutaryl coenzyme A (HMG-CoA) reductase activity and, hence, lowering the total cholesterol levels. Studies have shown that both BSO and its constituent THQ decrease mean arterial blood pressure, decrease heart rate in hypertensive rats, and promote cardiac health [10].

From the physicochemical point of view, RMP is a poorly water-soluble drug with poor oral bioavailability of 28-35% [7,11]. Thus, poor water solubility is one of the major predicaments limiting effective oral delivery of RMP. Similarly, the active THQ is a poorly water-soluble bioactive compound that shows poor bioavailability upon oral administration [12]. However, THQ present in BSO is inert and, thus, can be intact (solubilized form) when blended with a surfactant as a self-nanoemulsifying drug delivery system (SNEDDS) formulation. Accordingly, developing a combined liquid SNEDDS comprising RMP and BSO could lead to several formulations and therapeutic benefits. BSO could serve as an oil solubilizer (to enhance the loading and solubility of RMP), as a natural source of THQ, and as adjuvant synergistic therapy to treat hypertension and relative cardiovascular diseases alongside RMP.

Recently, bioactive self-nanoemulsifying drug delivery systems (BIO-SNEDDSs) have been adopted by the formulation scientists as a new vehicle to combine the benefits of natural bioactive oils along with the outstanding formulation benefits of SNEDDSs [3,13,14,15]. However, to the best of our knowledge, no previous study has explored the combination of RMP and BSO within SNEDDS and/or BIO-SNEDDS formulations. The BSO-loaded BIO-SNEDDS could potentially enhance the poor aqueous solubility and dissolution of both RMP and THQ.

Accordingly, the aim of the present study was to formulate an RMP SNEDDS using the bioactive BSO as the oil component of the delivery system. Several self-emulsification studies were conducted to select the most suitable cosolvent/surfactant for the formulation. Subsequently, an experimental design was constructed to optimize the RMP/THQ SNEDDS in terms of apparent solubility, droplet size, zeta potential, along with RMP and THQ dissolution profiles.

## 2. Results

### 2.1. Ultra-Performance Liquid Chromatography (UPLC) for Quantification of RMP and THQ

**Figure 1:**
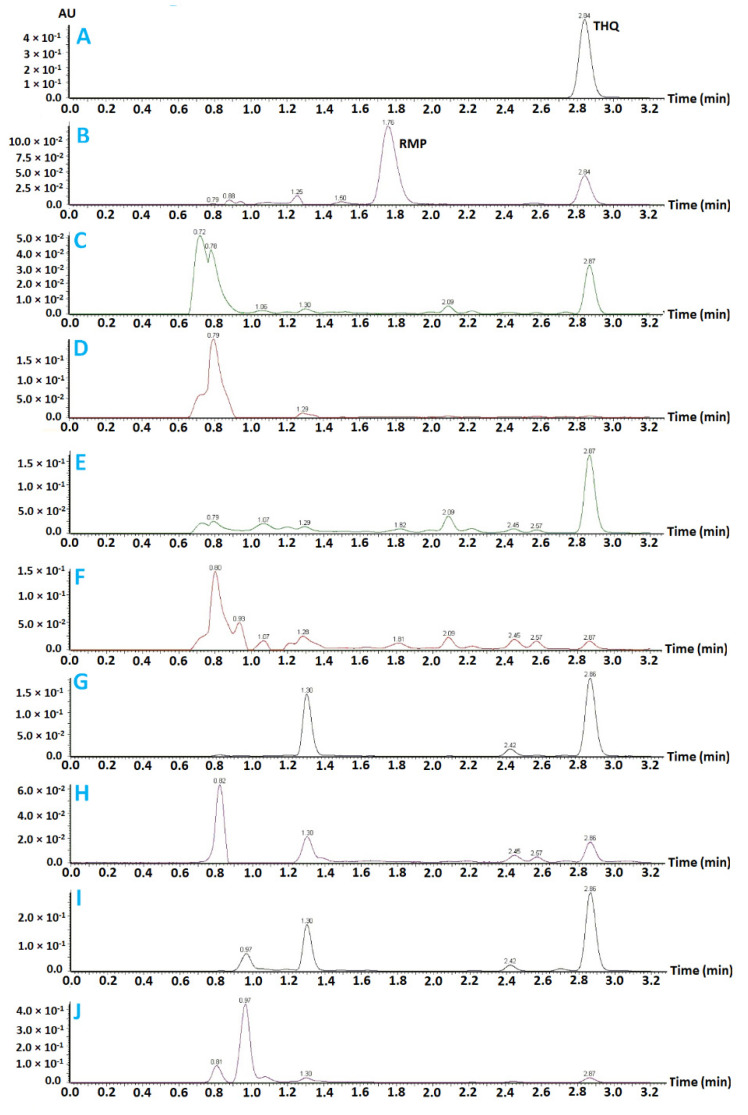
Forced degradation studies of THQ and RMP. (**A**): represents freshly prepared standard THQ solution (41.4 ppm), (**B**): freshly prepared standard RMP solution (50 ppm), (**C**): THQ acid hydrolysis, (**D**): RMP acid hydrolysis, (**E**): THQ base hydrolysis, (**F**): RMP base hydrolysis, (**G**): THQ thermal degradation, (**H**): RMP thermal degradation, (**I**): THQ oxidative degradation, and (**J**): RMP oxidative degradation.

An ultra-performance liquid chromatography method adapted from the USP assay was implemented, with minor modifications, for the quantification of RMP and THQ in aqueous and lipid-based systems. The forced degradation studies revealed that the RMP and THQ peaks were well resolved from one another in the chromatograms. The developed method provided an efficient elution of the RMP peak (at 1.76 min) and the THQ peak (at 2.84) (Figure 1A,B), with excellent resolution from one another. In addition, the forced degradation studies showed that all degradation peaks were well resolved from the intact RMP and THQ peaks, except for the base hydrolysis (Figure 1E) study, which showed a small degradation peak at 1.82 min. Furthermore, the developed method showed excellent linearity (R2 > 0.99) over the ranges for RMP (1.50–50.00 ppm) and for THQ (0.12–41.40 ppm).

### 2.2. Self-Emulsification Assessment

In total, 16 lipid-based formulations were selected for the screening process. The formulations were screened by varying their components, using one oil, two cosurfactants, one cosolvent, and eight surfactants to select the optimal formulation components with the strongest potential to develop efficient RMP/THQ SNEDDS (Table 1). The study showed that high BSO proportions (35%) were not mutually miscible with several cosurfactants and surfactants (F1–F7). Interestingly, formulations containing the surfactant SR-P80 were able to show good mutual solubility even at higher BSO proportions (up to 50%) (F8–F10). However, the formulations lacked acceptable homogeneity upon aqueous dilution (F10, poor homogeneity). Furthermore, the surfactants T85 and HCO-30 showed good miscibility and homogeneity with BSO. In particular, the formulations containing HCO-30 showed good miscibility, homogeneity, and clarity even with higher proportions of BSO (up to 50%) and different cosurfactants/cosolvents (F12–F16) (Table 1). The study showed that black seed oil (BSO) produced good-quality SNEDDSs with a wide range of cosurfactant/surfactant combinations. In particular, BSO-based systems showed enhanced self-emulsification efficiency with the cosolvent (Transcutol P-TCP) and hydrogenated castor oil (HCO-30) (Table 1).

### 2.3. Experimentally Designed Phase Diagrams

#### 2.3.1. Model Analysis

A summary of the suggested RMP/THQ SNEDDS formulations (runs) and their corresponding actual responses is presented in Table 2. The Design-Expert software was utilized to statistically analyze the effects of formulation variables on each response separately, based on different types, including linear, 2FI, cubic, and quadratic. The linear and quadratic models showed high F-values, non-significant lack of fit, high adjusted and predicted R^2^ (difference < 0.2), and high adequate precision for the droplet size (R1), PDI (R2) and RMP apparent solubility (R4), respectively.(Table 3). Accordingly, they were selected as the optimal models for these corresponding responses (Table 3). On the other hand, zeta potential (ZP) value, along with the release of RMP and THQ at 15 min, showed low/NA F-values, low adjusted R^2^, negative predicted R^2^, and low/NA adequate precision (inadequate signal-to-noise ratio < 4).

#### 2.3.2. Droplet Size

**Figure 2:**
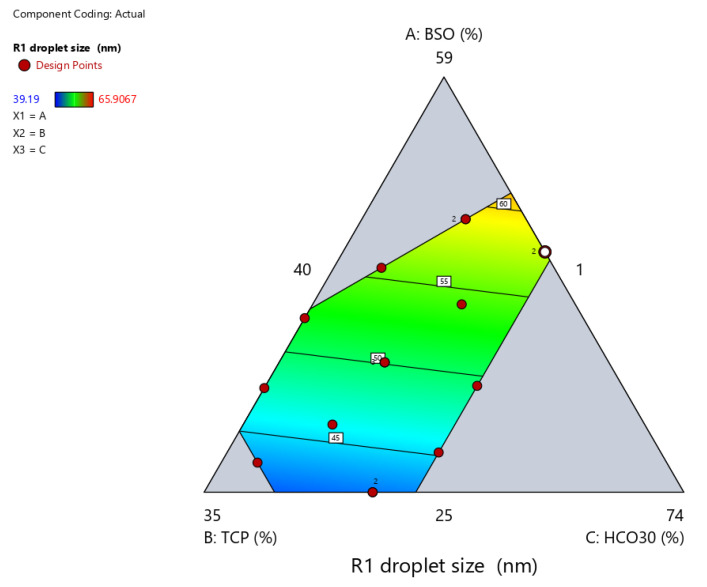
The contour representation of the effects of the formulation components on droplet size.

**Figure 3:**
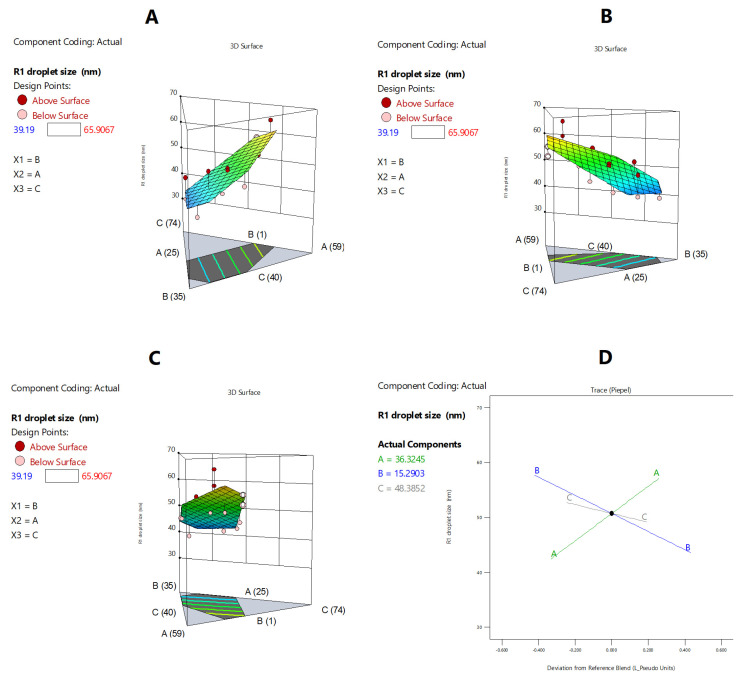
Graphical representation of the effects of the formulation components on droplet size: (**A**–**C**) The 3D surface representations of the effects of the proportions of BSO, TCP, and HCO-30, respectively, on droplet size. (**D**) Trace-type representation of the effects of the formulation components on droplet size. A: BSO % *w*/*w*; B: TCP % *w*/*w*; C: HCO-30 % *w*/*w*.

The data in Figure 2 show that the droplet size ranged from 39.2 to 65.9 nm within the tested design space. The correlations between the droplet size and independent formulation variables are presented in Figure 2 and Figure 3, Table 4, and Equation (1). The BSO proportion showed a strong positive effect on droplet size (*p* < 0.05) (Table 4). The droplet size significantly (*p* < 0.05) increased upon increasing the proportion of BSO in the formulation from 25% to 49.5% (Figure 3A,D). On the other hand, TCP showed a strong negative effect on droplet size (*p* < 0.05) (Table 4, Figure 3B,D). The droplet size significantly (*p* < 0.05) decreased upon increasing the proportion of TCP in the formulation, while the effect of HCO-30 was not significant (Table 4, Figure 3C,D). Accordingly, the droplet size of the formulation could be calculated from the final equation in terms of actual components (Equation (1)):

Droplet size (R1) = 0.948105 × BSO + 0.177766 × TCP + 0.280396 × HCO30(1)

#### 2.3.3. Apparent Solubility of RMP in Formulation

**Figure 4:**
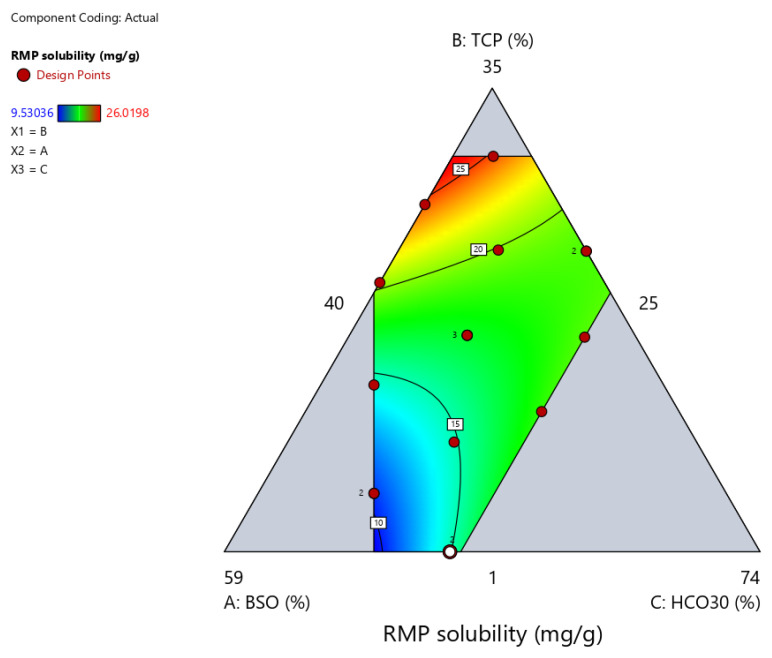
The contour representation of the effects of the formulation variables on RMP apparent solubility.

**Figure 5:**
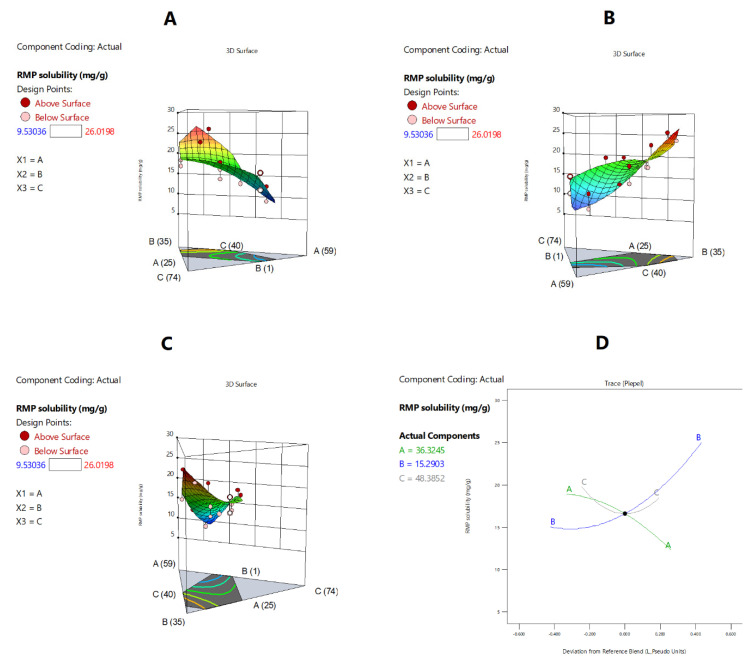
Graphical representation of the formulation components’ effects on RMP apparent solubility: (**A**–**C**) The 3D surface representations of the effects of the proportions of BSO, TCP, and HCO-30, respectively, on RMPapparent solubility. (**D**) Trace-type representation of the effects of the formulation components on apparent solubility. A: BSO % *w*/*w*; B: TCP % *w*/*w*; C: HCO-30 % *w*/*w*.

The apparent solubility of RMP ranged from 9.5 to 26.0 mg/g among the tested formulations (Table 2). The correlations between RMP apparent solubility and independent formulation variables are presented in Figure 4 and Figure 5, Table 5, and Equation (2). The proportion of BSO showed a strong negative effect on RMP apparent solubility (*p* < 0.05) (Figure 5A,D). The latter significantly (*p* < 0.05) decreased upon increasing the proportion of BSO in the formulation from 25% to 49.5%. On the other hand, TCP showed a strong positive effect on RMP apparent solubility (*p* < 0.05) (Figure 5B,D). The latter significantly (*p* < 0.05) increased upon increasing the proportion of TCP in the formulation, while the effect of HCO-30 was not significant (Figure 5C,D). Regarding the interaction effect of the binary components’ mixtures, the ANOVA test showed that the interaction of TCP × HCO-30 (BC) had a significant negative effect on the apparent solubility of RMP, while the remaining binary mixtures showed no significant effects (Table 5). Accordingly, RMP apparent solubility could be calculated from the final equation in terms of actual components (Equation (2)):

RMP apparent solubility (R3) = −0.0068 BSO + 2.3087 TCP + 1.1095 HCO30 + 0.0102 BSO × TCP −0.0183 BSO × HCO30 −0.0616 TCP × HCO30(2)

#### 2.3.4. Zeta Potential (ZP) and the Release of RMP and THQ at 15 Min

The data in Table 2 show that ZP varied from −15.3 to −36.7 mV among the tested formulations. The release of RMP (at 15 min) ranged from 58.1% to 95.8%, while that of THQ (at 15 min) ranged from 42.6% to 95.7% (Table 2). However, the ANOVA results showed no significant correlation between any of these responses and the independent formulation variables. In addition, the model for these responses showed very low adjusted R^2^ and negative predicted R^2^ (Table 3).

#### 2.3.5. Optimization of the SNEDDS

The droplet size, PDI and RMP apparent solubility showed significant model F-values and *p*-values, implying that the models were significant relative to the noise. On the other hand, the lack-of-fit F-value and *p*-value imply that the lack of fit is not significant relative to the pure error, confirming the good fit of the model. In addition, the models showed good agreement between predicted and adjusted R^2^, along with high adequate precision (signal-to-noise ratio > 4) (Table 3). Accordingly, the models of droplet size, PDI and RMP apparent solubility are suitable for navigating the design space.

In contrast, the model F-value and *p*-value of zeta potential, RMP release (%) and THQ release (%) at 15 min imply that these models are not significant relative to the noise (Table 3). In addition, these models showed very low adjusted R^2^ and negative predicted R^2^. The adequate precision was low (inadequate signal-to-noise ratio < 4), which implies that this model should not be used to navigate the design space. Accordingly, no valid correlations could be computed for these responses, and the overall mean could be used to represent them.

According to these findings, the software was utilized to optimize the SNEDDS formulations according to the following requirements: minimize droplet size, PDI, and ZP (i.e., the ZP should be highly negative and towards the −36.7 limit value), and maximize the apparent solubility of RMP, THQ amount in the formulation, and the release of RMP and THQ at 15 and 60 min. Accordingly, the optimized formulation BSO/TCP/HCO-30 (32.25/27.75/40) was suggested for further studies.

#### 2.3.6. Validation of the Experimental Model

**Figure 6:**
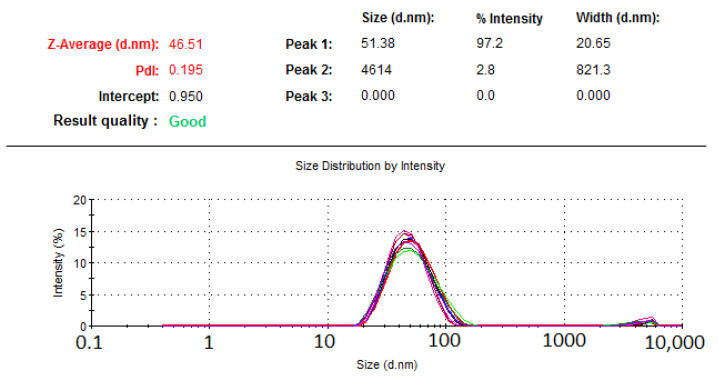
Droplet size and polydispersity index (PDI) of the optimized formulation, as measured by photon correlation spectroscopy.

The droplet size, PDI, and apparent RMP solubility of the optimized formulation were found to be 47 nm, 0.2, and 25 mg/g, respectively (Table 6, Figure 6). The actual findings of the optimized formulation were compared against the expected droplet size, PDI, and apparent solubility of RMP (Table 6). The optimized formulation parameters were close to the predicted values of responses, and fell within 95% prediction intervals. Accordingly, the experimental model was successfully validated for the droplet size, PDI, and apparent solubility of RMP. In addition, the optimized formulation showed a high negative ZP value, as well as high release % of RMP and THQ at 15 min.

### 2.4. In Vitro Dissolution

**Figure 7:**
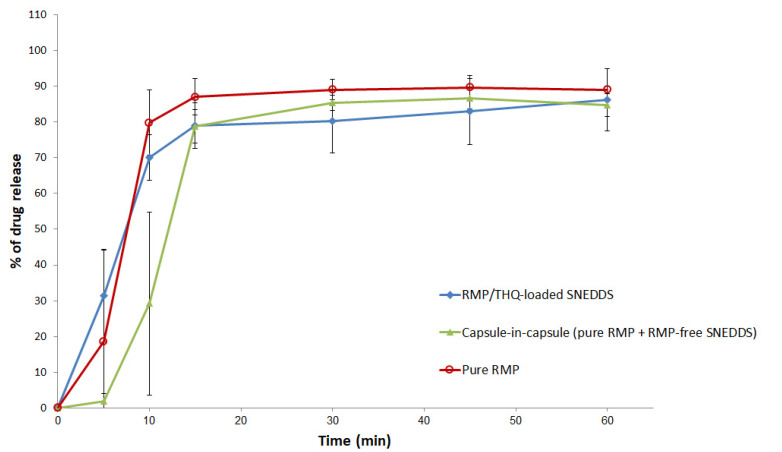
In vitro dissolution of RMP SNEDDS formulations at pH 1.2. Data are represented as the mean ± SD, *n* = 3.

The pure RMP, RMP/THQ-loaded SNEDDS, and the combination of pure RMP + RMP-free SNEDDS (capsule-in-capsule) showed enhanced dissolution of RMP, with a maximum of 89.7, 86.2, and 86.7% RMP release, respectively (Figure 7). The combination of pure RMP + RMP-free SNEDDS (capsule-in-capsule) showed an initial delay in drug release, but it showed >84% RMP release by the end of the dissolution study. However, no significant differences in DE% were found between these formulations.

**Figure 8:**
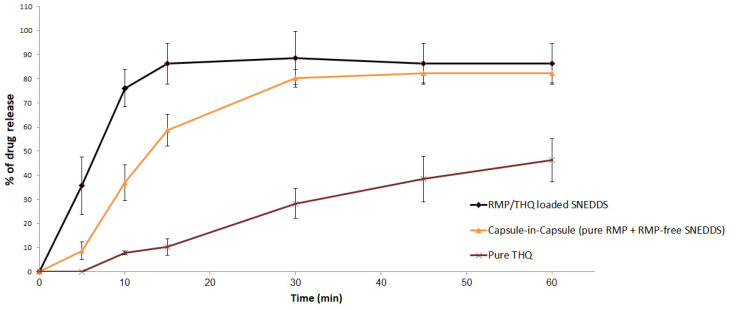
In vitro dissolution of THQ SNEDDS formulations at pH 1.2. Data are represented as the mean ± SD, *n* = 3.

On the other hand, pure THQ showed poor dissolution behavior, with a maximum of 46% release within 60 min. Meanwhile, the THQ SNEDDSs (RMP-loaded and RMP-free) showed significantly higher THQ release, with a maximum of 88.7% and 82.4%, respectively (*p* < 0.05 for DE%) (Figure 8).

### 2.5. Accelerated Stability Study

**Figure 9:**
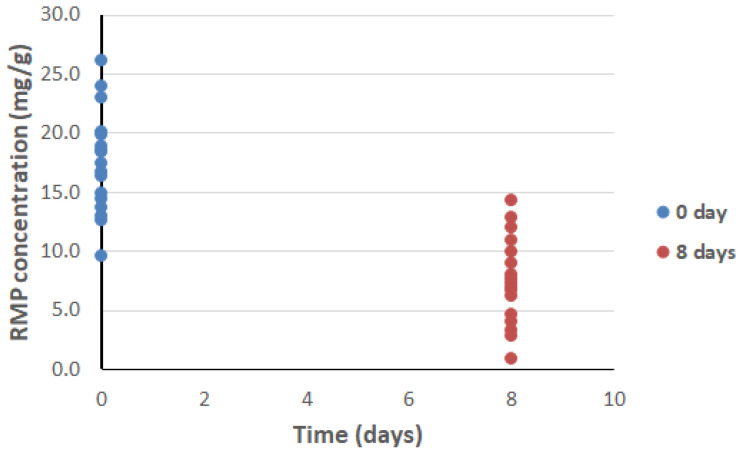
Accelerated RMP stability study of drug-loaded SNEDDSs.

The initial apparent solubility of RMP in drug-loaded SNEDDSs ranged from 9.5 to 26.0 mg/g (Figure 9). After storage for 8 days, RMP concentrations in the SNEDDSs were significantly (*p* < 0.05) reduced, to the range of 0.9–14.2 mg/g.

## 3. Discussion

Solubility is the driving force for absorption, and adequate drug solubilization in the intestinal fluid is a necessity for achieving sufficiently high oral drug bioavailability [16]. Poor drug solubility is one of the main hurdles in the drug discovery and development process. Accurate prediction of solubility remains very challenging, and there is an immense need for independent benchmarking and continuous enhancement of the present in silico models [17].

In the present study, BSO was used within all of the RMP formulations to serve as a bioactive oil vehicle of the SNEDDSs, as a natural source of THQ, and as adjuvant synergistic therapy for RMP to treat hypertension and related cardiovascular diseases.

Although the formulation of SNEDDSs appears to be comparatively simple, the selection of the formulation excipients and their relative proportions in the formulation is very complex. Only very specific pharmaceutical excipient combinations can lead to effective self-emulsifying systems [18]. Within the current study, a standard assessment method was adopted to assess the self-emulsification efficiency of the formulations based on four parameters, namely; the mutual miscibility, spontaneity, homogeneity, as well as physical appearance [18]. Formulations containing 50% surfactant were used extensively in the current study. These formulations were not essentially the most efficient self-emulsifying systems for each excipients combination, however they represented a common reference for each system and warranted that all of the formulations were efficiently dispersed to form systems [19]. According to the adopted assessment criteria, the formulation was accepted as an SNEDDS only if it exhibited complete excipient miscibility, along with good spontaneity and homogeneity. Semi-clear and clear physical appearances were preferred because they reflect a high chance that the developed emulsions are within the nano size range.

Most of the surfactants with higher (>12) HLB values (e.g., T20, T80, Kr-El, Kr-RH40) showed poor miscibility with BSO and, therefore, failed to meet the acceptance criteria of the self-emulsification efficiency (Table 1, F1-F7). Interestingly, SR-P80 (super-refined polysorbate 80) was the only highly hydrophilic surfactant that showed good miscibility with BSO, which could be due to its highly purified nature (Table 1, F8–F10). However, the latter experienced oil-phase separation upon adding high (50%) proportions of BSO to the formulations (Table 1, F10). Meanwhile, the less hydrophilic surfactants (HLB ≤ 11, e.g., HCO-30 and T85) showed good miscibility with BSO, along with good spontaneity and homogeneity (Table 1, F11–F16). In particular, combinations of HCO-30 and BSO showed excellent self-emulsifying properties and more transparent physical appearance, with different cosurfactant/cosolvent ratios. On the other hand, the preliminary solubility study showed that TCP presented the highest RMP solubility. Accordingly, the BSO/TCP/HCO-30 system was selected as containing the optimal excipients for RMP SNEDDS formulations, and was further investigated in the subsequent phase diagram studies.

Current approaches to developing self-nanoemulsifying drug delivery systems (SNEDDSs) involve using the “trial and error approach”, which involves changing one parameter at a time, or the conventional “ternary phase diagrams” technique. These methods are mostly empirical, tedious, and costly [20,21,22,23]. Therefore, in the present study, we utilized the experimental design for a time-effective and accurate optimization of SNEDDS phase diagrams [22].

The experimental design was utilized to evaluate the influence of formulation variables on dependent responses, compute actual equations for numerical prediction of important responses, and achieve a balanced optimization among different formulation attributes, including apparent drug solubility, droplet size, zeta potential, and drug release for both RMP and THQ.

**Figure 10:**
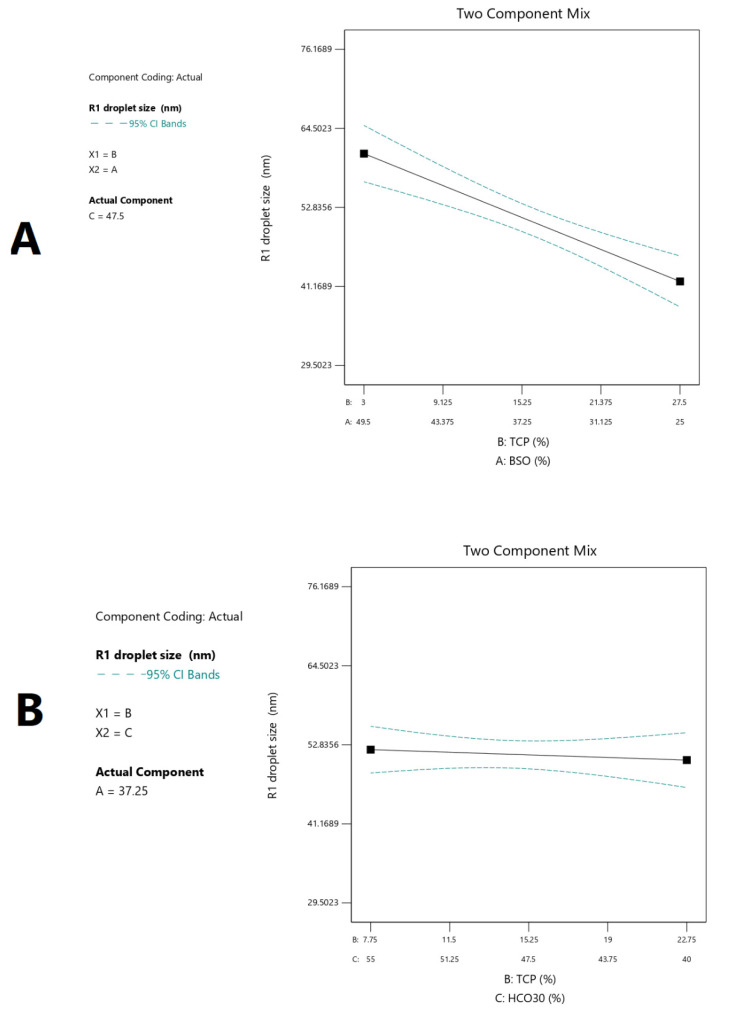
Two-component design graph of the effects of cosolvent TCP proportion on droplet size in case of maintaining the (**A**) surfactant proportion and (**B**) oil proportion at constant levels.

Among the screened responses, the droplet size (linear model) and apparent solubility of RMP (quadratic model) showed good accuracy and significance. The droplet size of the formulation upon aqueous dilution is crucial in self-emulsification, as it can influence the rate and extent of drug release [24]. The results revealed that the droplet size was significantly (*p* < 0.05) influenced by the proportions of the oil (BSO) and cosolvent (TCP) in the formulation. Increasing the proportion of BSO (*w*/*w* %) induced a linear increase in the droplet size. These findings are strongly consistent with previous studies that showed increased droplet size upon increasing the proportion of oil [24,25], and could be attributed to an increase in hydrophobicity and/or the corresponding reduction in the amount of surfactant in the formulation [24,26]. On the other hand, there was a linear decrease in the droplet size upon increasing the proportion of the cosolvent TCP (from 1 to 30% *w*/*w*) in the formulation. Previous studies showed controversial findings regarding the effect of cosolvent proportion on formulation droplet size. Valicherla et al. reported increased droplet size upon increasing the proportion of TCP from 10% to 30% [27]. Alghananim et al. reported that increasing the cosurfactant (Transcutol HP)/surfactant ratio decreased the region of nanoemulsion formation [28]. In contrast, Yoo et al. reported that droplet size decreased upon increasing the proportion of the cosurfactant Transcutol HP from 5 to 15%, after which the droplet size increased instead [29]. In the present study, the design was restricted to maintain the surfactant proportion ≥ oil proportion. Hence, increasing the cosolvent proportion would be predominately at the expense of the oil proportion, increasing the ratio of water-soluble excipients (i.e., surfactant and cosolvent) over water-insoluble excipients (i.e., oil), which could be responsible for the observed reduction in droplet size. This explanation could be represented by the change from a semi-clear appearance (F14) to a clear appearance (F15) upon increasing the TCP% from 0 to 15% (Table 1). The analysis from the experimental design confirmed this fact, as the droplet size steeply decreased upon increasing the TCP%, while the surfactant ratio was constant (Figure 10A). On the other hand, droplet size showed a negligible change upon increasing the TCP%, while the oil ratio remained constant (Figure 10B). In contrast to previous studies [24,28], the present study reports that the surfactant proportion had no significant effect on the droplet size. This finding could be due to the fact that (i) the design was constrained to keep the surfactant proportion ≥ oil proportion, and/or (ii) the tested surfactant range was narrow (40–55%).

On the other hand, the apparent solubility was significantly (*p* < 0.05) influenced by the oil (BSO) and cosolvent (TCP) proportions in the formulation. Increasing the oil (BSO) proportion (*w*/*w* %) resulted in a decrease in RMP apparent solubility, while the latter increased when increasing the TCP proportion in the formulation. These findings are in good agreement with previous studies that showed maximized RMP solubility in pure cosolvents and limited solubility in pure oils [30]. These findings could be related to the intermediate value of RMP octanol partition coefficient (log *p* = 3.32) [31], which implies low solubility in lipids and greater solubility in amphiphilic cosurfactants and cosolvents [32]. These data reveal that RMP is a hydrophobic rather than a lipophilic moiety. Therefore, it might not a suitable candidate for Type I, II, or IIIA lipid formulation classification system (LFCS) systems, which contain substantial amounts of lipophilic materials [33].

On the other hand, the experimental models for ZP and for RMP and THQ release showed inconsistent results and inadequate precision; hence, they could not be used to navigate the model space. The inconsistent release of RMP and THQ among different model runs could be due to gel formation upon exposure of the formulations to aqueous media, which lasted for >30 min in some formulations (Table 2) and, therefore, could be the reason behind the delayed and inconsistent release of RMP and THQ.

The optimized formulation BSO/TCP/HCO-30 (32.25/27.75/40) showed high apparent solubility of RMP (25.5 mg/g), good THQ content (2.7 mg/g), nanometric (47 nm) droplet size, acceptable PDI (0.2), and good (−30.13 mV) ZP values. These findings are strongly correlated with previous studies that showed low droplet size and acceptable ZP values with SNEDDSs containing HCO-30 and TCP [30]. The low formulation droplet size is highly desirable, and could be strongly associated with efficient self-emulsification, as well as rapid and enhanced drug release from the formulation [30]. The surface charge on droplets (represented by zeta potential) plays a vital role in the physical stability of nanoemulsions. If the droplets exhibit a high negative or positive zeta potential value, they should repel one another (reduce droplet agglomeration), which is expected to enhance dispersion stability [34]. The optimized formulation containing the non-ionic surfactant HCO-30 at 40% *w*/*w* exhibited a relatively high negative zeta potential (−30.1 mV). These findings could be attributed to the presence of some anionic impurities in the surfactant (such as free fatty acids), or to the adsorption of anionic species from the water (such as hydroxyl ions) to the droplet surfaces [35,36].

As per the current dissolution data, pure RMP showed >85% release within 15 min. Previous studies showed controversial results of pure RMP dissolution. Singh et al. reported that pure RMP showed a maximum of 24% dissolution up to 180 min dissolution at pH 1.2 [37]. Alhasani et al. reported that pure RMP showed a maximum of 59% drug release up to 120 min dissolution at pH 1.2 [30]. On the other hand, Zaid et al. reported that RMP showed a high water solubility/dose ratio; RMP tablets showed >85% release within 15 min at pH 1.2, 4.5, and 6.8, suggesting the biowaiver eligibility of the lower-strength ramipril immediate-release tablets [38,39]. These discrepancies might occur due to one or more of the following reasons: (1) differences in tested RMP strength, (2) differences in dissolution volume, and/or (3) differences in pure RMP particle size between different studies.

Most importantly, our in-vitro dissolution studies revealed that the RMP/THQ-loaded SNEDDS showed good release of RMP and THQ, which was similar to that of pure RMP (*p* = 0.237) and significantly higher than that of pure THQ (*p* < 0.05). The superior enhancement of drug dissolution in the case of SNEDDS formulations could be attributed to the ability of SNEDDSs to provide a favorable nanoemulsion environment to keep the drug solubilized within the nano-sized micelles formed upon exposure of the formulation to GI fluids.

Interestingly, the isolation between RMP and SNEDDS formulations (maintained by capsule-in-capsule dosage form) showed no significant adverse effect on the release of RMP (*p* = 0.065). These findings are quite important, because the present study showed significant RMP degradation within drug-loaded liquid SNEDDSs (Figure 9), which is also consistent with the findings of previous relevant studies [30]. Therefore, the combined pure RMP + RMP-free SNEDDS (capsule-in-capsule) could be a potential dosage form combining the solubilization benefits of SNEDDSs along with enhancing RMP stability through its isolation from lipid-based excipients during storage.

Future research directions should involve in vivo animal disease modeling and pharmacokinetic/pharmacodynamics studies to investigate the effect of the combined RMP/THQ formulation on blood pressure. In addition, comprehensive accelerated and long-term stability studies should be conducted to evaluate the impact of RMP isolation in RMP capsule-in-capsule SNEDDSs on the stability of RMP and THQ within the formulation.

## 4. Materials and Methods

### 4.1. Plant Material and Extraction of Bioactive Oils

#### 4.1.1. Collection and Extraction of Seeds

The seeds of *Nigella sativa* (*N. sativa*) Linn. (Black seeds), of the family Ranunculaceae, were collected from the central part of Bangladesh in the month of March. In total, 500 g of seeds was cleaned with fresh water and sundried to remove any moisture. Then, the seeds were cold-pressed, and the oil was filtered and stored in a screw-capped amber glass bottle for further use [3].

#### 4.1.2. BSO Standardization

Thymoquinone (THQ)—one of the principal bioactive constituents of *N. sativa* from its volatile oil—was used to standardize BSO. THQ stock solution (414 μg/mL) was used as a reference solution for the standardization of BSO. Serial THQ concentrations (0.12–41.40 μg/mL) were prepared, and the actual amount of THQ in BSO was calculated based on the THQ calibration curve. Accurately 9–13 mg of BSO was separately dissolved in 1.8 mL of solvent within 2 mL Eppendorf tubes and analyzed by UPLC. The amount of THQ present in BSO was found to be 9.6 ± 0.5 mg/g.

### 4.2. Chemical and Reagents

Ramipril (RMP) was purchased from Jai Radhe Sales (Ahmedabad, India). Thymoquinone (THQ) was purchased from Sigma-Aldrich (St. Louis, MO, USA). Imwitor 988 (I988), Imwitor 308 (I308), Kolliphor EL (KrEL), high-purity Kolliphor EL (KrEL HP), and Kolliphor RH40 (Kr-RH40) were purchased from BASF (Ludwigshafen, Germany). Hydrogenated castor oil (grade HCO30) was supplied by Nicole chemical Co., (Tokyo, Japan). Super-refined Tween 80 (SR-T80) was obtained from CRODA (Dusseldorf, Germany). Tween 85 (T85) and Tween 20 (T20) were supplied by Merck-Schuchardt OHG (Germany) and BDH (England), respectively. The cosolvent Transcutol^®^ P (TCP) (purified diethylene glycol monoethyl ether) was supplied by Gattefossé (Lyon, France). HPMC capsules (size 2) and fish gelatin capsules (size 00) were donated by Capsugel (Greenwood, SC, USA). All other reagents were of analytical grade and used without further purification.

### 4.3. Ultra-Performance Liquid Chromatography (UPLC) for Quantification of Ramipril

An ultra-performance liquid chromatography method adapted from the USP assay was implemented, with minor modifications, for the simultaneous quantification of RMP and THQ within aqueous and lipid-based systems. The method involved preparing a 0.1% *w*/*v* sodium lauryl sulfate solution (pH adjusted to 2.4 ± 0.1). Subsequently, the SLS solution was mixed with acetonitrile at a 45:55 ratio (*v*/*v*) (pH adjusted to 2.75 ± 0.1, solution A). The assay involved a reversed-phase UPLC method. The mobile phase composition was solution A:acetonitrile (67:33 % *v*/*v*), and the runtime was 3.2 min. Peak separation was achieved using an Acquity^®^ UPLC HSS T3 (2.1 × 100 mm, 1.8 µm) column connected with an Acquity guard filter, and the flow rate was maintained at 0.25 mL/min. To ensure maximum selectivity, RMP and THQ were quantified at two different wavelengths, namely, at 210 and 254 nm, respectively, with an injection volume of 2 µL. Furthermore, forced degradation studies (such as acid-based hydrolysis, alkaline-based hydrolysis, and thermal stress conditions) were performed to ensure efficient resolution of both RMP and THQ from their degradation peaks.

The developed method provided efficient elution of a sharp RMP peak with good linearity over the range 1.5–50.0 ppm (R2 > 0.99), which proved to be efficient for the analysis of RMP in lipid-based formulation systems.

### 4.4. Preparation of Drug-Free Liquid SNEDDSs

Liquid SNEDDSs were initially prepared using the bioactive oil (BSO) with a non-ionic surfactant, cosolvent, and/or cosurfactant. The produced mixture was efficiently homogenized (vortexed for ≈1 min) to achieve maximum miscibility [13]. The prepared mixtures were then stored in 1.5 mL Eppendorf tubes for further characterization.

### 4.5. Self-Emulsification Assessment

A visual test to assess the self-emulsification properties reported above was modified and adopted in the present study [19]. The visual test was mainly designed to measure the apparent spontaneity of emulsion formation against time. Formulation was subjected to 1:1000 aqueous dilution in a glass beaker at room temperature (RT), and the contents were stirred for 5 min.

The self-emulsification test was a combined test used for the evaluation of the excipients’ miscibility, formulation spontaneity, homogeneity, and appearance after aqueous dilution, as follows:

- The blends of different excipients—such as mixtures of oils, surfactants, and/or cosolvents—were examined carefully to evaluate the mutual miscibility between the components.
- The spontaneity of the formulation was judged as “good” when the droplets easily spread in water, to form an emulsion, within 1 min. It was judged as “moderate” when the droplets took 1–5 min to completely spread in water. Finally, the formulation was judged as “poor” when the droplets tended to coalesce, needed high shear mixing, and/or took >5 min to completely spread in water.
- The homogeneity of the formulation was judged as “good” when the formulation was able to be dispersed in water without causing any phase separation. It was judged as “poor” when the formulation resulted in phase separation and/or oil floating upon aqueous dilution.
- For further characterization, the appearance of the formulations was also judged as turbid, bluish (semi-clear), or clear according to the degree of clarity of the formulation after aqueous dilution.
- Descriptive green, yellow, and red colors were utilized to easily distinguish between good, moderate, and poor performance of the formulations. According to the assessment criteria, the formulation was accepted as an SEDDS/SNEDDS only if it showed complete excipient miscibility, as well as at least moderate spontaneity and homogeneity.

### 4.6. Experimentally Designed Phase Diagrams

The phase diagrams were constructed using experimental design (Design-Expert^®^, version number 13, Stat-Ease, Inc., (Minneapolis, MN, USA)) to reduce the number of runs, obtaining comprehensive analysis of the data in a time-effective manner [22,40]. A 17-run custom design was generated to estimate a special cubic model for the three independent formulation variables (Table 7) [24]. The design involved no blocks, and the study included three independent variables: the proportions of oil (represented by BSO % *w*/*w*, A), cosolvent (represented by TCP %*w*/*w*, B), and surfactant (represented by HCO-30 % *w*/*w*, C) (Table 8). Based on preliminary self-emulsification and apparent solubility data, the range of each variable (%) was selected as follows:

25 ≤ BSO ≤ 49.5;

1 ≤ TCP ≤ 30;

40 ≤ HCO-30 ≤ 55;

(HCO-30) − (BSO) ≥ 0;

Total components = 100.

The amount of the formulation was kept constant (1000 mg), while the ratio of the three variables was varied. Among several responses (i.e., dependent variables), the following were represented: R1: droplet size after 2 h of aqueous dilution of drug-free formulation (nm); R2: zeta potential after 2 h of aqueous dilution of drug-free formulation (mV); R3: solubility of RMP in the SNEDDS (mg/g); R4: RMP release (%) at 15 min; R5: THQ release (%) at 15 min.

Seventeen SNEDDS formulations were prepared, as presented in Table 2. The correlation of factors with response variables was then fitted into different mathematical models (i.e., quadratic, cubic, or special cubic) [24]. Analysis of variance (ANOVA) was applied to determine the significance of each design model, as well as the independent variables and their interactions [27]. For each response, the optimal model was selected, showing a high correlation coefficient, a high F-value, a non-significant lack of fit, high adjusted and predicted R2 (difference < 0.2), and high adequate precision [40]. Afterward, a desirability function using Design-Expert (version number 13, Stat-Ease, Inc., (Minneapolis, MN, USA) was applied to optimize factors for desirable responses. The suggested optimized formulations were prepared and considered as a checkpoint to evaluate the accuracy of the design. The predicted values of each response were determined and compared to their corresponding actual values.

### 4.7. Apparent Solubility of RMP in SNEDDSs

Preliminary studies showed rapid degradation of RMP in SNEDDS formulations. To minimize drug degradation, the formulations were freshly prepared by incorporating RMP in the lipid-based formulations in excess amounts. An excess amount of the drug was rapidly dissolved in the formulation by vortexing (for ≈1 min), followed by sonication (375H, Jencons Scientific Ltd, Bedfordshire, England) at room temperature (20 ± 2 °C) for up to 1 h. To separate the undissolved drug particles, the mixtures were centrifuged using a benchtop centrifuge (PrO-Research K2015, Centurion Scientific Ltd., Chichester, UK) at 10,000 rpm for 5 min [41]. The supernatant (approximately 15 mg) was diluted in 1.8 mL of acetonitrile within 2.0 mL Eppendorf tubes [42]. Subsequently, samples were analyzed by the adopted assay method using UPLC.

### 4.8. Droplet Size and Zeta Potential

The formulations were diluted at a ratio of 1:1000 *v*/*v* (formulation: distilled water) and mixed for 1 min. After 2 h, the mean droplet size, distribution, and polydispersity index (PDI) of the diluted formulations were measured using a Zetasizer Nano ZS analyzer (Model ZEN3600, Malvern Panalytical Ltd., Malvern, UK)). The particle size of the aqueous dispersions was evaluated by photon correlation spectroscopy (PCS) using a dynamic light scattering (DLS) mode at 25 °C. The zeta potential of each formulation was evaluated using laser Doppler velocimetry (LDV) mode at 25 °C [13,22].

### 4.9. Preparation of the Optimized SNEDDS

The optimal SNEDDS was prepared by mixing the oil, surfactant, and cosolvent at an optimized ratio. RMP was loaded in the SNEDDS formulation at a concentration of 10 mg/g (*w*/*w*). Finally, the components were thoroughly mixed and sonicated for 1 h to ensure complete drug solubilization and homogenization [43].

### 4.10. In Vitro Dissolution Studies

**Figure 11:**
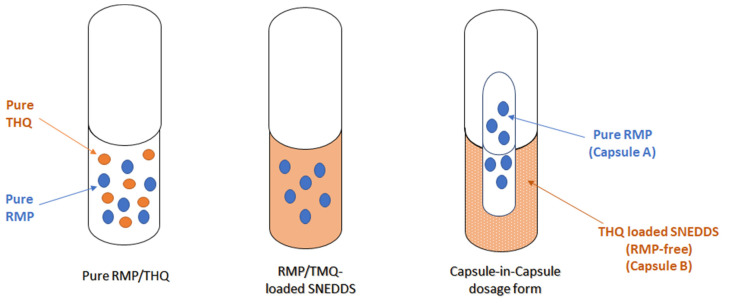
A schematic diagram of RMP/THQ dosage forms.

The in vitro dissolution studies were conducted to compare the dissolution profile of pure RMP/THQ against those of the RMP-loaded SNEDDS and the combination of the pure RMP + RMP-free SNEDDS (capsule-in-capsule). A schematic diagram of the three dosage forms is represented in Figure 11. The pure RMP/THQ and RMP-loaded SNEDDS (equivalent to 2.5 ± 0.1 mg of RMP and 0.7 ± 0.1 mg of THQ) were used to fill fish gelatin capsules (size 00). To prepare the pure RMP + RMP-free SNEDDS (capsule-in-capsule) dosage form, pure RMP was used to fill small capsule A (HPMC size 2 inner capsule), and the RMP-free SNEDDS was used to fill large capsule B (fish gelatin size 00 outer capsule). Subsequently, the smaller capsule A was inserted into capsule B to form the RMP capsule-in-capsule dosage form. A few turns of nonreactive wire helix were attached to each capsule to prevent it from floating in the dissolution medium [44].

The in vitro dissolution studies were conducted according to the USP-NF “Ramipril Capsules” monograph (USP 43–NF 38), with minor modifications. According to the monograph, the tests were conducted using USP dissolution apparatus II (Model: UDT-804, LOGAN Inst. Corp., Franklin, NJ, USA) coupled with a paddle stirrer at a speed of 50 rpm. Then, 500 mL of simulated gastric fluid (with no enzymes, pH = 1.2) was used as the dissolution medium, and the temperature was maintained at 37 ± 0.5 °C [42,45]. To compare the dissolution profiles of different formulations, the dissolution time was extended for 60 min. The samples were withdrawn at 5, 10, 15, 30, 45, and 60 min and filtered using a filter syringe, before being analyzed via the UPLC method. The dissolution studies were carried out in 3 replicates, and the dissolution efficiency (DE)% was utilized to compare the drug release from different formulations [13,46].

### 4.11. Accelerated Stability Study

At the initial time point, an excess amount of RMP was dissolved in the formulation by vortexing (for ≈1 min), followed by sonication (375H, Jencons Scientific Ltd., Bedfordshire, UK) at room temperature (20 ± 2 °C) for up to 1 h. Then, the mixtures were centrifuged, and the supernatant (approximately 15 mg) was diluted in 1.8 mL of acetonitrile within 2.0 mL Eppendorf tubes and analyzed by UPLC. Subsequently, the samples were stored at room temperature (20 ± 2 °C) for up to 8 days. Samples were withdrawn and reanalyzed after an 8-day interval, and the degradation of RMP was evaluated by the changes in RMP concentration [47].

### 4.12. Statistical Analysis

One-way analysis of variance (ANOVA) followed by post hoc tests (LSD) (IBM SPSS Statistics 26) was used to compare the dissolution results (in terms of DE%). A paired *t*-test was used to evaluate the RMP stability. A *p*-value < 0.05 was considered to be statistically significant [13]. Grubbs’ test (using the QuickCalcs feature in GraphPad Software website) was used to detect outliers at the significance level alpha = 0.05.

## 5. Conclusions

RMP—an important medication for hypertension and heart failure—combined with BSO containing THQ, was successfully formulated to overcome the poor aqueous solubility of RMP/THQ and the significant degradation of RMP in lipid-based formulations. The optimized formulation showed relatively high drug loading, low droplet size, and high negative zeta potential. Most importantly, the optimized formulation showed enhanced release of RMP and THQ at pH 1.2, whether in the form of an RMP-loaded SNEDDS or the combination of pure RMPand an RMP-free SNEDDS. The latter system introduces a potential dosage form that could offer enhanced release of RMP and THQ, along with potential enhancement of RMP stability within the formulation.

## Figures and Tables

**Table 1:** Self-emulsification assessment of RMP/THQ-containing lipid-based formulations.

Formulation Code	Oil	Cosurfactant			Surfactant								Self-Emulsification Assessment				
	BSO	I308	I988	TCP	SR-P80	Kr-EL	Kr-EL (HP)	T80	T20	Kr-H40	T85	HCO30	Miscibility	Time (min)	Appearance	Homogeneity	Overall Assessment
F1	35	15				50							IM	-			X
F2	35		15			50							IM	NA			X
F3	35			15		50							IM	NA			X
F4	35	15					50						IM	NA			X
F5	35	15						50					IM	NA			X
F6	35	15							50				IM	NA			X
F7	35	15								50			IM	NA			X
F8	35	15			50								M	<1	T	H	√
F9	45	10			45								M	<1	T	H	√
F10	50				50								M	<1	T	OF	X
F11	35	15									50		M	1–2		H	√
F12	35	15										50	M	<1	C	H	√
F13	45	10										45	M	<1	SC	H	√
F14	50											50	M	<1	SC	H	√
F15	35			15								50	M	1–2	C	H	√
F16	45			10								45	M	<1	SC	H	√

BSO: black seed oil, I308: Imwitor I308, I988: Imwitor I988, TCP: Transcutol P, SR-P80: super-refined Tween 80, Kr-EL: Kolliphor EL, T80: Tween 80, T20: Tween 20, Kr-H40: Kolliphor RH40, T85: Tween 85, IM: immiscible, M: miscible, C: clear, SC: semi-clear, T: turbid, H: homogenous, OF: oil-floating, √: accepted, and X: rejected. Green, yellow and red background represent good, moderate and poor performance, respectively.

**Table 2:** Suggested RMP/THQ SNEDDS formulations and their corresponding actual responses.

Run	Independent Variables				Dependent Variables (Responses)						
	A: BSO (%)	B: TCP (%)	C: HCO-30 (%)	R1: Droplet Size (nm)	R2: Polydispersity Index (PDI)	R3: Zeta Potential (mV)	R4: Apparent RMP Solubility (Day 0) (mg/g)	R5: RMP Release (%) at 15 min	R6: THQ Release (%) at 15 min	R7: Apparent RMP Solubility (Day 10) (mg/g)	R7 Formulation Gelling at 30 min
1	47.4	5.3	47.4	65.9	0.213	−25.5	9.5	72.8	65.7	4.0	No
2	33.7	11.3	55.0	46.3	0.229	−17.4	20.0	58.1	42.6	6.7	No
3	25.0	23.0	52.0	41.4	0.237	−32.7	17.4	89.0	77.6	12.8	Yes
4	35.6	16.9	47.5	51.5	0.189	−19.7	14.4	71.0	73.4	7.6	Yes
5	25.0	23.0	52.0	48.9	0.220	−15.3	18.8	75.8	65.5	10.9	No
6	30.5	23.1	46.3	52.4	0.200	−25.0	22.9	70.0	70.4	8.0	No
7	33.5	26.5	40.0	46.8	0.202	−21.6	26.0	67.7	63.0	11.9	Yes
8	28.3	16.7	55.0	43.0	0.258	−31.3	19.8	82.9	88.3	7.2	No
9	40.4	9.0	50.6	50.7	0.184	−22.5	13.6	74.7	62.9	6.2	Yes
10	35.6	16.9	47.5	50.7	0.213	−21.0	18.3	77.0	82.9	6.8	Yes
11	39.3	20.7	40.0	47.6	0.150	−21.5	18.5	80.6	86.3	8.9	Yes
12	43.4	13.2	43.4	56.1	0.148	−23.4	14.9	82.4	77.1	4.6	No
13	47.4	5.3	47.4	60.6	0.139	−27.2	13.0	65.4	78.9	0.9	Yes
14	27.4	30.0	42.6	39.2	0.230	−24.8	23.9	95.8	90.1	14.2	No
15	35.6	16.9	47.5	49.6	0.208	−15.4	16.6	85.5	95.7	9.9	No
16	44.7	1.0	54.3	54.2	0.196	−19.1	12.5	62.8	67.0	2.8	Yes
17	44.7	1.0	54.3	57.6	0.192	−36.7	16.3	82.9	82.3	3.2	Yes

**Table 3:** ANOVA analysis of the measured responses for the selected models.

Responses	Selected Model	F-Value	*p*-Value	Lack of Fit *p*-Value	Adjusted R2	Predicted R2	Adequate Precision
R1 droplet size	Linear model	17.86	0.0001	0.2740	0.6782	0.5676	10.284
R2 polydispersity index (PDI)	Linear model	13.40	0.0005	0.8693	0.6079	0.4675	8.969
R3 ZP	Mean model	NA	NA	0.9416	0.0000	−0.1289	NA *
R4 apparent solubility of RMP	Quadratic	11.85	0.0004	0.5338	0.7722	0.6222	11.562
R5 RMP release at 15 min	Linear model	1.55	0.2460	0.4732	0.0646	−0.2835	3.4337
R6 THQ release at 15 min	Mean	NA	NA	0.2543	0.0000	−0.1289	NA *

NA: not available; (*) case(s) with leverage of 1.0000: predicted R² and PRESS statistic not defined.

**Table 4:** Formulation components’ effects (Peipel) on droplet size.

Component	Gradient in Reals	Component Effect	Gradient Standard Error	Approx. t for H₀ Gradient = 0	Prob > |t| (*p*-Value)	Gradient in Pseudo
A-BSO	73.24	14.74	14.38	5.09	0.0002	24.90
B-TCP	−48.63	−14.10	11.46	−4.24	0.0008	−16.53
C-HCO30	−23.79	−3.56	19.99	−1.19	0.2537	−8.09

**Table 5:** Analysis of variance (ANOVA) of the quadratic model presenting the correlation between independent formulation variables and RMP apparent solubility.

	Sum of Squares	df	Mean Square	F-Value	*p*-Value	
**Model**	253.72	5	50.74	11.85	0.0004	Significant
Linear mixture ⁽*⁾	189.21	2	94.60	22.08	0.0001	
AB	3.48	1	3.48	0.81	0.3866	
AC	1.42	1	1.42	0.33	0.5766	
BC	31.23	1	31.23	7.29	0.0207	
**Residual**	47.12	11	4.28			
Lack of fit	25.09	6	4.18	0.95	0.5338	Not significant
Pure error	22.03	5	4.41			
**Total correlation**	300.84	16				

⁽*⁾: Inference for linear mixtures uses type I sums of squares. A: BSO % *w*/*w*; B: TCP % *w*/*w*; C: HCO-30 % *w*/*w*.

**Table 6:** Validation of the experimental design model.

Response	Predicted Mean	n	95% PI Low	Data Mean	95% PI High	Data SD
R1 droplet size *	46.73	3	40.30	47.47	53.14	0.99
R2 polydispersity index (PDI) *	0.189	3	0.156	0.203	0.222	0.010
R3ZP	−23.54	4	−30.502	−30.13	−16.57	2.28
R4 apparent solubility of RMP *	25.37	4	21.47	25.57	29.28	4.43
R5 RMP release at 15 min	81.93	3	66.14	78.91	97.73	6.33
R6 THQ release at 15 min	74.68	3	57.40	86.22	91.95	8.48

* represent acceptabe models for navigating the design space.

**Table 7:** The characteristics of the experimental design model.

File Version	13.0.11.0		
**Study type**	Mixture	**Subtype**	Randomized
**Design type**	I-optimal (coordinate exchange)	**Runs**	17.00
**Design model**	Special cubic	**Blocks**	No Blocks

**Table 8:** The design variables’ constraints and coding.

Variable	Name	Units	Minimum	Maximum	Mixture Component Coding	Coded Low	Coded High	Mean	Standard Deviation
A	BSO	%	25	47.36	L_Pseudo	+0 ↔ 25	+0.72 ↔ 49.5	36.32	7.53
B	TCP	%	1	30	L_Pseudo	+0 ↔ 1	+0.85 ↔ 30	15.29	8.77
C	HCO30	%	40	55	L_Pseudo	+0 ↔ 40	+0.44 ↔ 55	48.39	4.98

## Data Availability

Data is contained within the article.
